# High pressure phase transitions of paracelsian BaAl_2_Si_2_O_8_

**DOI:** 10.1038/s41598-019-49112-1

**Published:** 2019-09-02

**Authors:** Liudmila A. Gorelova, Anna S. Pakhomova, Sergey V. Krivovichev, Leonid S. Dubrovinsky, Anatoly V. Kasatkin

**Affiliations:** 1 Department of Crystallography, Institute of Earth Sciences, St. Petersburg State University, University Emb. 7/9, 199034, Saint Petersburg, Russia; 2 Deutsches Elektronen-Synchrotron (DESY), Petra III, Notkestraße 85, 22607 Hamburg, Germany; 3 Kola Science Centre, Russian Academy of Sciences, Fersman str. 14, 184209 Apatity, Russia; 4 Bayerisches Geoinstitut, University of Bayreuth, Universitätsstraße 30, 95447 Bayreuth, Germany; 5 Fersman Mineralogical Museum of the Russian Academy of Sciences, Leninskiy pr. 18, 2, 119071 Moscow, Russia

**Keywords:** Solid-state chemistry, Chemical physics, Mineralogy

## Abstract

Three new polymorphs of aluminosilicate paracelsian, BaAl_2_Si_2_O_8_, have been discovered using synchrotron-based *in situ* high-pressure single crystal X-ray diffraction. The first isosymmetric phase transition (from paracelsian-I to paracelsian-II) occurs between 3 and 6 GPa. The phase transition is associated with the formation of pentacoordinated Al^3+^ and Si^4+^ ions, which occurs in a stepwise fashion by sequential formation of Al-O and Si-O bonds additional to those in AlO_4_ and SiO_4_ tetrahedra, respectively. The next phase transition occurs between 25 and 28 GPa and is accompanied by the symmetry change from monoclinic (*P*2_1_/*c*) to orthorhombic (*Pna*2_1_). The structure of paracelsian-III consists of SiO_6_ octahedra, AlO_6_ octahedra and distorted AlO_4_ tetrahedra, i.e. the transition is reconstructive and associated with the changes of Si^4+^ and Al^3+^ coordination, which show rather complex behaviour with the general tendency towards increasing coordination numbers. The third phase transition is observed between 28 and 32 GPa and results in the symmetry decreasing from *Pna*2_1_ to *Pn*. The transition has a displacive character. In the course of the phase transformation pathway up to 32 GPa, the structure of polymorphs becomes denser: paracelsian-II is based upon elements of cubic and hexagonal close-packing arrangements of large O^2−^ and Ba^2+^ ions, whereas, in the crystal structure of paracelsian-III and IV, this arrangement corresponds to 9-layer closest-packing with the layer sequence **ABACACBCB**.

## Introduction

Local coordination of atoms in crystalline compounds is an important characteristic that determines their physical properties and behaviour under changing thermodynamic conditions. Being ‘useful fiction’, the parameters of coordination polyhedra can be used to estimate the physical properties of minerals by summation of properties of separate polyhedra^1,2^. Indeed, individual coordination polyhedra have the same properties invariant from structure to structure (such as shape, size, heat capacity, thermal expansivity, compressibility, etc.)^3,4^. Thus, the phase transitions accompanied by the changes in the coordination of chemical elements are associated with drastic changes of their chemical and physical properties. Silicates are probably the most important materials in human history, due to their leading role in geology, mineralogy, construction industry, pottery, material science, etc. The crystal chemistry of silicates is traditionally based upon the idea of nearly exclusive four- (under ambient conditions)^5^ or sixfold (under high pressure)^6,7^ coordination of silicon. Over the last few years there have been several reports on the high-pressure phase transitions of crystalline silicates, that were accompanied by the formation of new coordination environments around Si atoms and, in particular, SiO_5_ square pyramids or trigonal bipyramids. The first observation of this kind was made by Angel *et al*.^8^, who described a high-pressure modification of CaSi_2_O_5_ featuring Si atoms in five- and sixfold coordinations. This study initiated considerable theoretical work on the diversity of hypothetic phases with pentacoordinated silicon^9–13^, but very few experimental confirmations have been reported till recently, when a number of experimental results have appeared in the literature^14–18^.

Similarly to silicon, aluminum occurs predominantly in tetrahedral or octahedral coordination, though fivefold trigonal-bipyramidal polyhedra are much more common compared to silicon in silicates^19^. The well-known example is andalusite, Al_2_SiO_5_^20^, which contains Al in octahedral and trigonal-bipyramidal coordinations. The AlO_5_ trigonal bipyramids are also present in the crystal structures of different compounds, including PbCa_2_Al_8_O_15_^21^, Ba_8_Al_10_B_12_O_41_^22^, LiAl_7_B_4_O_17_^23^, NaAl_2_(AlSi_3_)O_10_(OH)_2_^24^, Tl_3_Al_2_P_3_O_12_^25^, Al_6_Ti_2_O_13_^26^, KAl_2_(PO_4_)_2_(OH)·*n*H_2_O^27,28^, Al_2_(OH)_3_(VO_4_)^29^, Al_4_B_2_O_9_^30^, Al_5_BO_9_^31^ (note that the last two compounds contain Al in all three coordination).

In our recent high-pressure studies on feldspar-related minerals such as danburite, CaB_2_Si_2_O_8_^15^, and hurlbutite, CaBe_2_P_2_O_8_^32^, we have discovered polymorphs of these materials, featuring *T*O_5_ trigonal bipyramids and *T*O_6_ octahedra (*T* = Si, Be, P). Observations of these exotic structural units has motivated us to investigate high-pressure behavior of closely related mineral paracelsian, BaAl_2_Si_2_O_8_^33^, in order to illuminate correlations between transformation pathways and chemical and topological properties. Paracelsian and its monoclinic modification celsian^34^ possess aluminosilicate framework structures and belong to the feldspar group. Despite this fact, the framework topology of paracelsian is different from the feldspar topology. Paracelsian has a pseudo-orthorhombic crystal structure with the *P*2_1_/*a* symmetry^33,35,36^. Its overall topology is identical to those observed in danburite, CaB_2_Si_2_O_8_, and hurlbutite, CaBe_2_P_2_O_8_^32^, but the ordering scheme of tetrahedral cations is different. In paracelsian and hurlbutite, chemically distinct tetrahedral cations are in perfect alternation, whereas, in danburite, there are double Si_2_O_7_ and B_2_O_7_ tetrahedral groups^15^. The high-pressure behavior of celsian was studied recently by Curetti *et al*.^37^, who discovered a new *P*2_1_/*c* high-pressure modification existing above 5.7 GPa.

In general, crystalline materials with the BaAl_2_Si_2_O_8_ composition attract considerable attention due to their various technological applications. They are widely used in glass and ceramic industries, including the production of low temperature co-fired ceramic (LTCC) materials^38,39^, due to the low dielectric constants and good microwave dielectric properties^40–44^. As BaAl_2_Si_2_O_8_ has low thermal expansion coefficients, high melting point and corrosion resistance, it is used as a refractory material^40,45–48^. In addition, celsian-based compounds (see below) are used as environmental barrier coating and matrix material in fibre-reinforced composites^49,50^. The BaAl_2_Si_2_O_8_ materials are also of importance as phosphors. For instance, BaAl_2_Si_2_O_8_ doped by Eu^2+^, Eu^3+^, Sm^2+^, Sm^3+^, Dy^3+^, Li^+^, Tb^3+^, Ce^3+^ demonstrate promising luminescent properties^51–56^.

From the mineralogical point of view, compounds with the BaAl_2_Si_2_O_8_ stoichiometry belong to the feldspar group, members of which are important rock-forming minerals found in all types of rocks and constituting over 50% of the Earth’s crust. They are essential constituents of most igneous rocks, but also found in association with metamorphic and sedimentary rocks. Feldspar-group minerals are very important for petrogenetic processes and any model used to describe phase equilibria and the structural changes of minerals in the Earth’s crust requires knowledge of their thermoelastic properties (such as the volume dependence upon pressure (the bulk modulus) and the volume thermal expansion, as well as their evolution with temperature and pressure). For instance, paracelsian, which is the subject of the current study, is associated with exhalative hydrothermal processes, low-and medium-grade metamorphism, and have been found in sedimentary and meta-sedimentary rocks.

Due to their high geological relevance and various technological applications, numerous X-ray diffraction studies at non-ambient conditions (high temperature (HT) and/or pressure (HP)) have been performed with detailed studies focused on orthoclase, sanidine, microcline, albite, and anorthite^57–72^. In feldspar-related minerals and inorganic compounds with divalent cations such as celsian, BaAl_2_Si_2_O_8_^37^, PbAl_2_Si_2_O_8_^64^, SrAl_2_Si_2_O_8_^73,74^, and solid solutions between these compounds^75–77^, several phase transitions were found upon compression. However, all the reported phase transitions occur with a symmetry change but without reconstructive transformations in the aluminosilicate frameworks. Herein we report on the results of a high-pressure single-crystal X-ray diffraction study of paracelsian, BaAl_2_Si_2_O_8_, the compound showing step-wise pressure-induced transitions with the formation of phases with Si and Al changing their coordinations from tetrahedral to octahedral through a pentacoordinated form.

## Experimental Section

In our experiments, we have used the samples of natural paracelsian from the Benallt Mine, Gwynedd, Wales, UK, obtained from a private systematic collection of Anatoly V. Kasatkin.

The high-pressure *in situ* single-crystal diffraction experiments were performed at the Extreme Conditions Beamline (ECB) at Petra III, DESY (Hamburg, Germany) using the procedures described previously^15,17,32^. For the pressure generation, the Mao-type symmetric diamond anvil cell (DAC) produced at ECB was used. Diamond anvils of Bühler-Almax type (X-ray opening 56 degree) with culet diameters of 300 μm were glued on tungsten backing seats and aligned. Rhenium gaskets were indented to about 30 μm and subsequently drilled to obtain sample chambers with approximate diameters of 150 μm. Two crystals with the approximate sizes of 10 × 10 × 5 μm^3^ were placed inside the sample chambers along with ruby sphere and tungsten crystals of about the same size. To achieve quasi-hydrostatic conditions, the DAC was loaded with a neon pressure-transmitting medium using the high-pressure gas loading systems of ECB. The pressures (Table 1) were determined using ruby fluorescence^78^.

**Table 1:** Crystallographic data for paracelsian polymorphs from the experiment.

Phase	Paracelsian-I			Paracelsian-II						Paracelsian-III	Paracelsian-IV
P, GPa	0.0001	0.14(1)	3.01(1)	6.84(1)	10.66(1)	14.22(1)	17.76(1)	21.25(1)	24.70(1)	28.50(1)	32.42(1)
Sp.gr.	*P*2_1_/*c*			*P*2_1_/*c*						*Pna*2_1_	*Pn*
*a*, Å	8.5756(1)	8.5663(2)	8.5385(2)	8.9406(3)	8.9359(2)	8.8971(3)	8.8683(3)	8.8425(4)	8.8068(4)	5.434(4)	8.742(3)
*b*, Å	9.5731(3)	9.5654(6)	9.4721(5)	9.0353(6)	8.9109(4)	8.8374(5)	8.7814(6)	8.7323(7)	8.6895(7)	8.743(3)	5.387(3)
*c*, Å	9.0681(3)	9.0579(5)	8.8015(5)	7.1777(6)	6.9954(5)	6.9042(5)	6.8155(7)	6.7210(8)	6.6556(8)	9.6849(19)	9.634(2)
β, °	90.1696(17)	90.183(3)	90.185(3)	90.243(4)	90.041(4)	90.095(4)	89.905(5)	89.868(6)	89.845(7)	90	91.35(2)
*V*, Å^3^	744.44(4)	742.21(7)	711.85(6)	579.81(6)	557.02(5)	542.86(5)	530.76(6)	518.96(8)	509.32(8)	460.1(4)	453.5(3)
*D*_calc._, g/cm^3^	3.35	3.36	3.50	4.30	4.48	4.59	4.70	4.81	4.90	5.44	5.50

Monochromatic X-ray diffraction experiments were performed at ECB using X-rays with a wavelength of 0.2905 Å. Diffraction patterns were collected using a Perkin Elmer 1621 detector. Before the experiment, the detector-to-sample distance was calibrated with CeO_2_ standard using the procedure implemented in the program Dioptas^79^. At each pressure, both a wide-scan and a stepped ω-scan were collected for each crystal. Wide-scans consisted of 40 s exposure during rotations of ±20° of the DAC. Step scans in the range ±28 degree were performed with individual exposures taken over 0.5° intervals to constrain the ω angle of maximum intensity of each peak. Collected diffraction images were analyzed using the program CrysAlis Pro©^80^. The SHELXL program package^81^ was used for all structural determinations.

The crystal structures of paracelsian were solved and refined at eleven pressure points (see Table S1), including ambient pressure. The crystal structures of paracelsian-I and II were refined with anisotropic displacement parameters at all pressures (0–21 GPa), except for the last pressure point (25 GPa). The crystal structure of paracelsian-III (28 GPa) was refined with anisotropic displacement parameters for all cations (Ba, Si, Al) and isotropic parameters for O, whereas the crystal structure of paracelsian-IV (32 GPa) was refined with anisotropic displacement parameters for Ba and some Si and Al atoms only.

The equation-of-state fits were performed with the *EoSFit7*c program^82^. The CRYSTAL14 software package was used to perform the solid-state DFT calculations^83^. The Peintinger−Oliveira−Bredow split-valence triple-ζ (pob-TZVP) basis sets^84^ were used for all atoms along with the hybrid Becke-3−Lee−Yang−Parr (B3LYP) functional. The electron-density distribution functions were calculated using experimentally observed geometries for each pressure point and analyzed using the *TOPOND14* software^85,86^ with respect to the properties of (3,−1) bond critical points^86^.

## Results

### Crystal structure of paracelsian under ambient conditions

**Figure 1:**
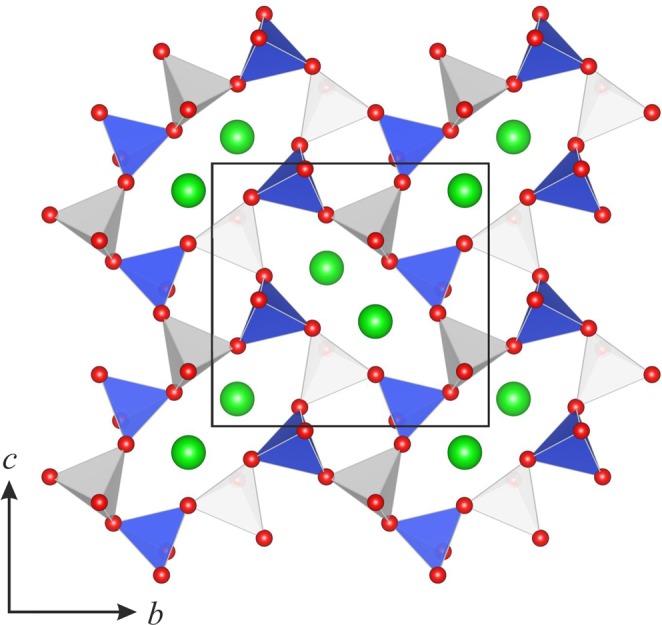
The crystal structure of paracelsian under ambient conditions, viewed along *a* axis. Blue and white tetrahedra represent AlO_4_ and SiO_4_, respectively. The Ba atoms are shown in green.

At ambient conditions the crystal structure of paracelsian was first determined by Smith^35^ as monoclinic but very nearly orthorhombic (*Pnam*). Later structure refinements^33,36^ confirmed the monoclinic character of the structure with the *β* angle in the range from 90.01° to 90.21°. The asymmetric unit contains four tetrahedrally coordinated cations *T* (two Si and two Al) atoms and eight O atoms. Polymerization of *T*O_4_ tetrahedra by corner sharing results in the formation of a tetrahedral framework, with channels outlined by four- and eight-membered rings along the *a* axis (Fig. 1). The channels with eight-membered rings are occupied by Ba atoms in either seven- (for Ba–O bonds shorter than 3 Å) or ninefold (taking into account two Ba–O bonds of 3.317(2) and 3.324(2) Å) coordination. The eight-membered rings are elliptically elongated with the ratio between the longest and shortest diagonals equal to 2.67.

As it was mentioned above, paracelsian is considered as a member of the feldspar group, though the topology of its tetrahedral framework is different from that in feldspars^35,87^. According to Smith and Brown^87^, the particular feature of the feldspar framework is the occurrence of double crankshaft chains cross-linked to form elliptical 8-membered rings. Such a description permits a simple explanation of the anisotropy of the physical properties of the material. The topological differences between feldspar and paracelsian structures^87^ could be described, using an algebraic code developed by Smith and Rinaldi^88^ in which a tetrahedron pointing up is denoted as **U** and one pointing down is denoted as **D**. According to this approach, eight-membered rings in the feldspar topology possess two types of configurations (**UUUUDDDD** and **DUUDUDDU**), whereas, in paracelsian, there is one type of eight-membered ring (**DUUDUDDU**) only.

### High-pressure phase transitions of paracelsian

**Figure 2:**
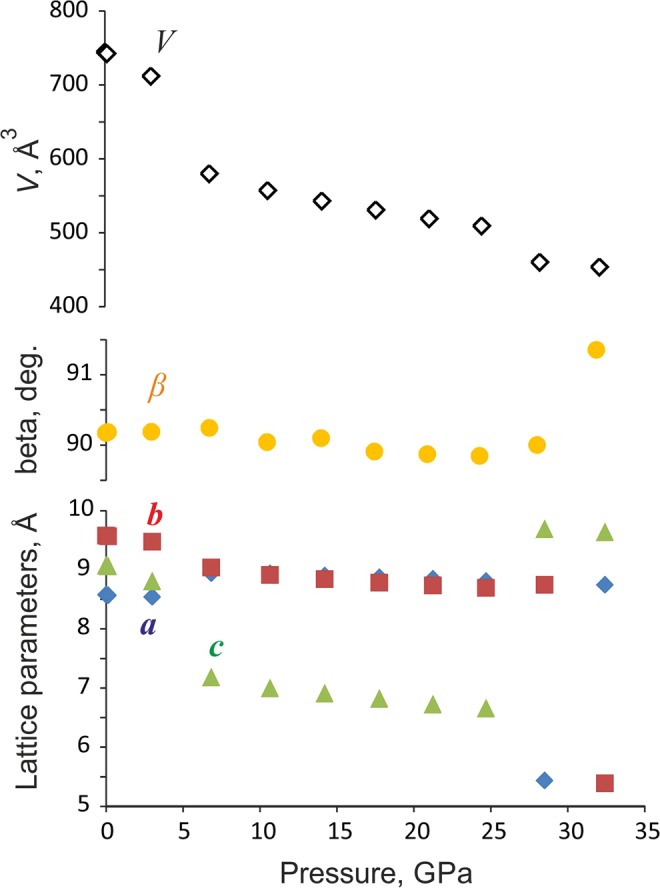
The evolution of the unit-cell parameters of paracelsian along compression. The errors are smaller than the size of the symbols.

The high-pressure behaviour of paracelsian was investigated up to 32 GPa by single-crystal synchrotron X-ray diffraction. Up to 3 GPa, a continuous contraction of the unit cell (Fig. 2, Table S1) and a shortening of interatomic distances (Table S2) are observed. The compression is anisotropic, with the *c* and *a* axes showing the highest and the lowest compressibility, respectively.

**Figure 3:**
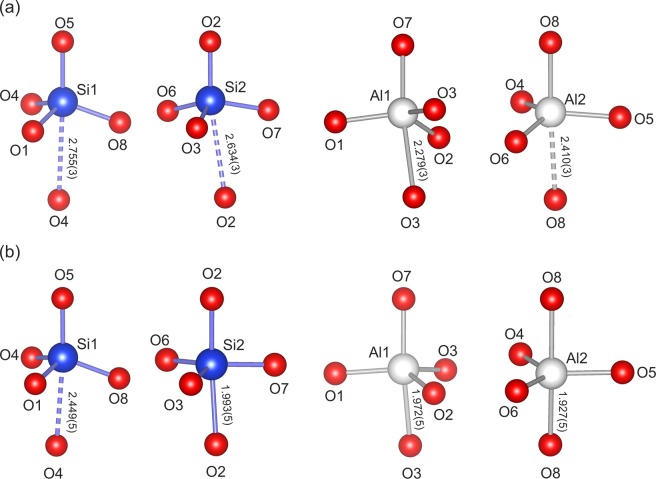
The *T*O_*n*_ (*T* = Si, Al; *n* = 4, 5) polyhedra at 7 (**a**) and 25 (**b**) GPa. The distances for other bonds at 7 GPa in silicon polyhedra are in the range 1.608(3)–1.642(3) Å, in aluminum polyhedra 1.702(3)–1.834(3) Å, at 25 GPa in silicon polyhedra 1.597(5)–1.706(6) Å and in aluminum polyhedra 1.712(5)–1.896(6) Å.

**Figure 4:**
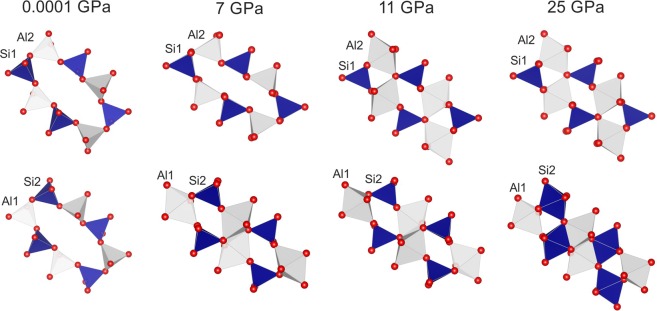
The evolution of the eight-membered rings of paracelsian and paracelsian-II along compression.

**Figure 5:**
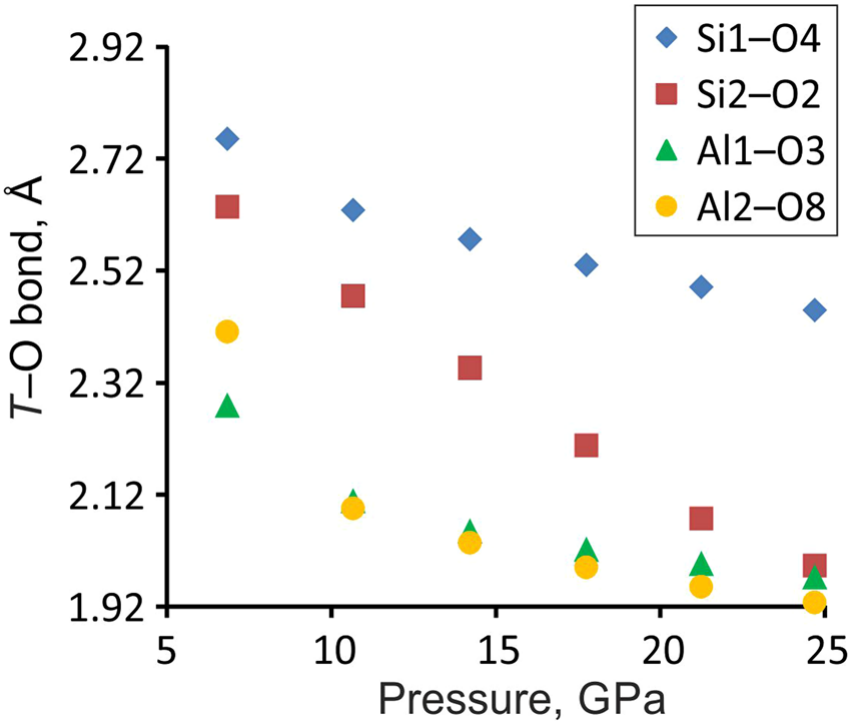
The evolution of the fifth bond in *T*O_5_ (*T* = Si, Al) polyhedra of paracelsian-II along compression.

**Figure 6:**
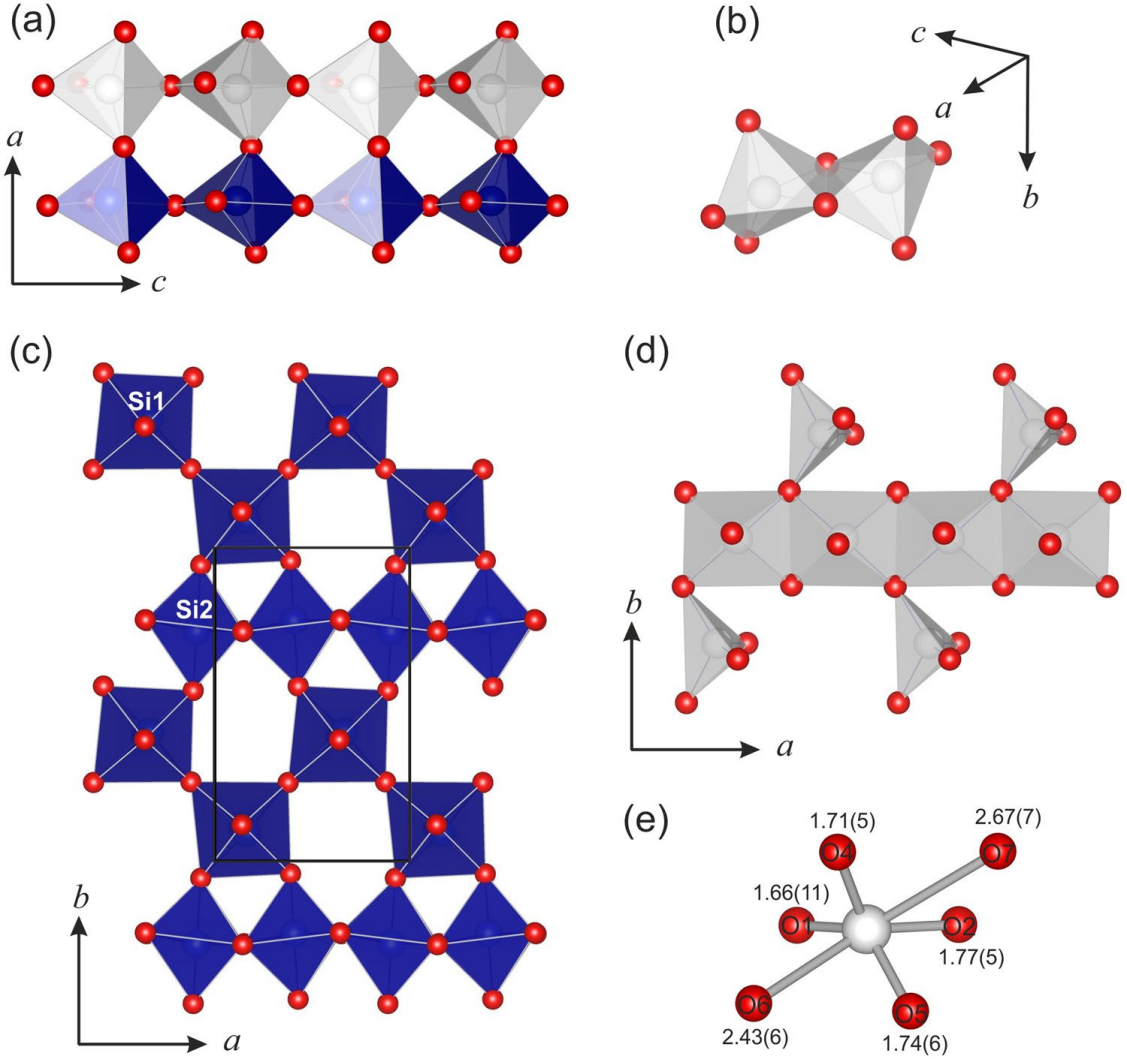
Fragments of crystal structures of paracelsian-II (**a**,**b**) at 25 GPa and paracelsian-III (**c**–**e**) at 28 GPa. Blue and white polyhedra represent SiO_*n*_ and AlO_*n*_ (*n* = 4, 5, 6), respectively. (**a**) chains of Al2O_5_ and Si2O_5_ trigonal-bipyramids; (**b**) Al_2_O_8_ dimer with Al1 central atom; (**c**) layer of SiO_6_ octahedra; (**d**) chain of Al1O_6_ octahedra and Al2O_4_ distorted tetrahedra; (**e**) coordination of Al2 atom.

Above 6 GPa, paracelsian-I (ambient-pressure modification) transforms into paracelsian-II, the first high-pressure phase that has the same monoclinic space group (*P*2_1_/*c*) as paracelsian-I. The phase transition is first-order, which is indicated by the abrupt change of the unit-cell parameters (Fig. 2, Table S1). The anomalous behaviour is observed for certain bond lengths (Table S2): the average 〈*T–*O〉 bond lengths in all *T*O_4_ tetrahedra increase between 3 and 7 GPa, which is an indication of increasing coordination number. The phase transition is induced by the rapid approach of additional oxygen atoms towards the coordination sphere of *T* atoms (Fig. 3a). The structural changes are displacive and are induced by shifts of the *T* (*T* = Si, Al) atoms along the *c* axis in such a way that across the eight-membered rings extra O2, O3 and O8 atoms approach coordination spheres of Si2, Al1 and Al2 atoms, respectively, (Fig. 4), leading to increase of their coordination numbers to fivefold. Between 3 and 7 GPa, the fifth Si–O distance decrease by more than 1.1 Å and the fifth Al–O distances decrease by more than 1.4 Å (Fig. 3a). At 7 GPa, the *T*O_4_ tetrahedra can be tentatively considered as *T*O_4+1_ configurations with the additional Si1–O and Si2–O contacts at distances of 2.755(3) and 2.634(3) Å, respectively, and the additional Al1–O and Al2–O contacts at distances of 2.279(3) and 2.410(3) Å, respectively. Upon further compression above 7 GPa length of the “fifth” contact in each *T*O_4+1_ polyhedron continuously decreases (Fig. 5). While the Al1–O8, Al2–O3 and Si2–O2 contacts undergo pronounced shortening and at 25 GPa their length become equal to 1.972(5), 1.927(5) and 1.993(5) Å, respectively, the Si1–O4 contact is more rigid and is longer than 2.4 Å at the same pressure (Fig. 5). At ~25 GPa, the geometry of three *T*O_5_ (where *T* is Si2, Al1 and Al2) polyhedra is trigonal-bipyramidal (Fig. 3b) with two long and three short equatorial *T*–O bonds (Fig. 3b, Table S2). The O–*T*–O apical bond angles are 7–12° away from the 180° angle required for a regular trigonal bipyramid. The formation of additional bonds results in the formation of dimers of edge-sharing *T*O_5_ polyhedra across the rings. However, the resulting structural units are different. The Si2O_5_ and Al2O_5_ groups share common vertices to form chains along the *c* axis (Fig. 6a), as it was observed for SiO_5_ polyhedra in danburite-II^15^, whereas Al1O_5_ bipyramids share the O–O edge to form a Al_2_O_8_ dimer (Fig. 6b), as found for datolite-II^17^. The coordination number of Ba atoms increases to 11 with the average bond lengths 2.71–2.85 Å depending upon the pressure. The BaO_11_ polyhedra link to each other by sharing faces to form chains extended along the *c* axis.

**Figure 7:**
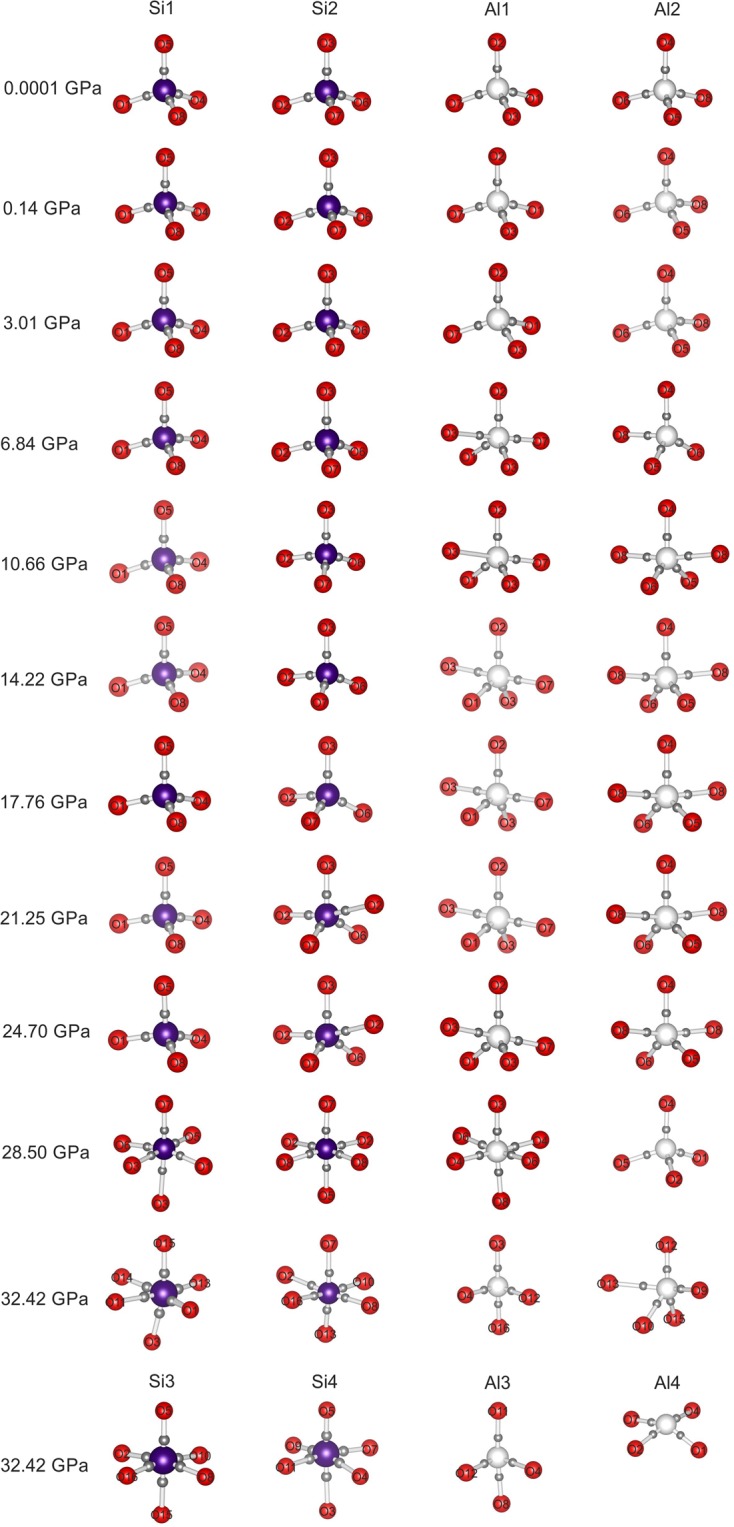
The SiO_*n*_ and AlO_*n*_ (*n* = 4, 5, 6) configurations in the crystal structure of paracelsian at different pressures in skeletal representations. The positions of BCPs are indicated with the small grey spheres.

The DFT calculations and the analysis of theoretical electron-density distributions at different pressures indicate the existence of the Al1–O3 bond (2.279(3) Å) at pressures above 7 GPa, the Al2–O8 bond (2.095(4) Å) at pressures above 11 GPa and the Si2–O2 bond (2.077(5) Å) at pressures above 21 GPa (Fig. 7, Table S3), which is manifested by the appearance of the (3,–1) bond critical point (BCP) for the respective atom pairs. If one accepts that the formation of a new bond indicates a phase transition, paracelsian-II can be separated into three phases, paracelsian-II*a*, paracelsian-II*b* and paracelsian-II*c*, which differ from each other by the presence of respective additional (fifth) *T–*O bonds. The transition between these polymorphs is continuous and isosymmetric, with paracelsian-II*a* and paracelsian-II*b* being intermediate phases between paracelsian-I and paracelsian-II*c*.

Upon compression above 28 GPa, another first-order phase transition is detected by abrupt change of unit cell parameters (Fig. 2). The new phase, paracelsian-III, has the *Pna*2_1_ space group (Table S1). Similarly to paracelsian-I and II, the crystal structure of paracelsian-III contains two Si, two Al, one Ba, and eight O sites. The *P*2_1_/*c* → *Pna*2_1_ transition is reconstructive and involves breaking of chemical bonds. As a result of the transition, the chains of Si2O_5_ trigonal bipyramids and isolated Si1O_4_ tetrahedra transform into sheets of SiO_6_ octahedra running parallel to the *ab* plane (Fig. 6c). In these layers, Si2O_6_ octahedra share edges to form chains parallel to the *a* axis that are connected to each other *via* zigzag chains of corner-sharing Si1O_6_ octahedra. The chains and dimers of AlO_5_ polyhedra present at 25 GPa transform into chains (Fig. 6d) of edge-sharing Al1O_6_ octahedra incrustated by strongly distorted Al2O_4_ tetrahedra (Fig. 6e). The coordination number of Ba increases from 11 to 12 with the average 〈Ba-O〉 bond length of 2.688 Å (Table S2). The presence of chemical bonds inside all polyhedra in paracelsian-III is in agreement with DFT and AiM calculations (Fig. 7, Table S3).

At the last pressure point of about 32 GPa, another phase, paracelsian-IV, is observed. It has the space group *Pn* and can be described as a distorted version of paracelsian-III. Due to the symmetry lowering (from orthorhombic to monoclinic), the number of crystallographic sites is doubled (Table S2), but no coordination changes are observed, which indicates a displacive character of the phase transition.

### Equation of state and compressibility of paracelsian-II

The obtained P-V data between 6 and 25 GPa were used to determine an equation of state of paracelsian-II. The initial volume V_0_ and the room-temperature isothermal bulk modulus K_T0_ were determined to 621(3) Å^3^ and 81(4) GPa, respectively, by fitting Birch-Murnaghan second-order equation of state. For paracelsian, paracelsian-III and -IV, the available pressure points were insufficient to constrain an equation of state.

## Discussion

High-pressure phases featuring SiO_5_ polyhedra are very rare in inorganic crystal chemistry and the mechanism of their formation is currently unclear. According to rules of thumb formulated by Prewitt and Downs^89^ and further generalized rules by Grochala *et al*.^90^, the increasing pressure leads to the increasing coordination numbers and the atomic arrangements in high-pressure structures show strong tendency to form close-packed arrays. According to Pakhomova *et al*.^15^, high-pressure crystal structures with pentacoordinated silicon may form as intermediate (transitional) configurations between relatively open ambient-pressure structure and ideal or distorted close-packed high-pressure structure.

**Figure 8:**
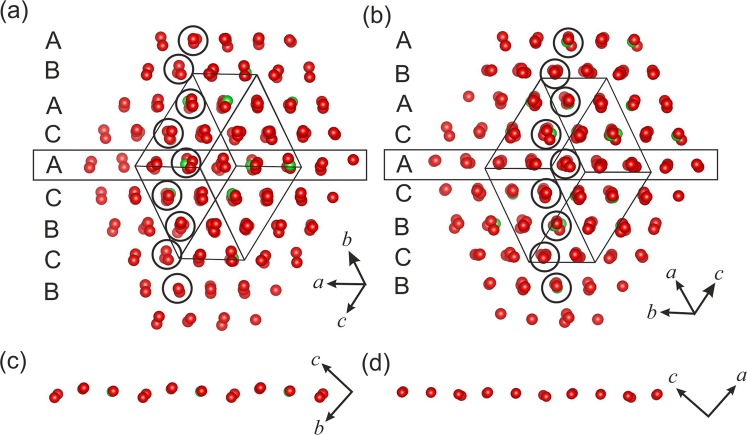
The evolution of close packing of oxygen atoms in the structure of paracelsian-III (**a**) and IV (**b**). The figures (**c**) and (**d**) demonstrate the **A**-layers of paracelsian-III and paracelsian-IV in the projection *bc* and *ac*, correspondingly.

Indeed, the arrangement of large ions (O^2−^ anions and *M*^2+^ cations (*M* = Ca, Ba) in the crystal structures of transitional phases (containing pentacoordinated Al, Si and P) of danburite, hurlbutite and paracelsian (i.e. danburite-II, hurlbutite-II, hurlbutite-III and paracelsian-II) are similar and can be described as having elements of distorted cubic and hexagonal close packings with a square-like contact^15^. However, the polymorphs of the three compounds at higher pressures possess geometrically different close-packed arrangements. The crystal structure of danburite, CaB_2_Si_2_O_8_ (danburite-III)^15^, is based upon the cubic (**ABC**) close packing of O^2−^ and Ca^2+^ ions with smaller cations in octahedral and tetrahedral interstices, whereas the crystal structures of hurlbutite-IV^32^ and paracelsian-III and IV are based upon more complex closest packing: 12-layer closest packing of Ca^2+^ and O^2−^ ions with the layer sequence **ABCACABCBCAB** and 9-layer closest packing of Ba^2+^ and O^2−^ ions with the layer sequence **ABACACBCB** (Fig. 8), respectively. The paracelsian III → paracelsian-IV phase transition is associated with small shifts of the packing ions, which results in more perfect close-packed arrangement. The same 9-layer closest packing was previously found for elemental Sm^91,92^ and 9*R*-modification of Li stable at low temperatures^93^ as well as for more chemically complex compounds such as ε-Hf_3_N_2_^94^. This Sm-type layer consequence had been considered as a combination of face-centered cubic and hexagonal close-packed structures with the ratio 1:2^95^.

In the course of high-pressure phase transitions in paracelsian, Al^3+^ ions adopt fivefold coordination first, before Si^4+^ cations do, which can be explained by the ionic radii (r_ion_) of Al^3+^ and Si^4+^, which are equal to 0.39 and 0.26 Å for tetrahedral coordination, respectively^96^. This prompts Al^3+^ ions to form additional bonds more easily than Si^4+^ ions. It is noteworthy that small B^3+^ cations in danburite (^tetr^r_ion_ = 0.11 Å) do not change their coordination at all and remain tetrahedrally coordinated under all observed high-pressure interval.

The previous high-pressure (up to 5 GPa) and high-temperature (up to 1100 °C) single crystal X-ray diffraction studies of feldspars revealed phase transitions governed by the changes in the values of bridging *T*–O–*T* angles between the rigid *T*O_4_ units, whereas the latter do not undergo significant distortion and compression/expansion. The phase transitions are therefore of displacive character, owing to the considerable flexibility of tetrahedral frameworks accompanied by the cooperative motions of tetrahedral groups, such as tilting and rotating^97,98^. Paracelsian, BaAl_2_Si_2_O_8_, demonstrates a contrasting behaviour: upon the compression the *T*O_4_ units undergo strong distortions with subsequent formation of *T*O_5_ trigonal bipyramids (for both Si and Al).

The information-based structural complexity of paracelsian have been calculated using the TOPOS program package^99^ following the procedure outlined by Krivovichev^100–102^. The isosymmetric paracelsian-I → paracelsian-II phase transition does not result in any changes of structural complexities (the information content per atom and per unit cell, *I*_*G*_ and *I*_*G*,*total*_, remain the same, 3.700 and 192.423 bits, respectively). The similar trend is observed for the paracelsian-II → paracelsian-III phase transition, whereas the formation of paracelsian-IV in the course of displacive phase transition (i.e. without changes in cation coordination) is associated with the increase in both *I*_*G*_ and *I*_*G*,*total*_ parameters (4.700 and 244.423 bits, respectively). This agrees well with the general empirical rule that high-pressure-induced reconstructive phase transitions (with coordination changes) do not result in the increase of structural complexity (not taking into account chemical bonds), whereas high-pressure-induced displacive transitions usually lead to the formation of structurally more complex phases.

## Conclusions

In conclusion, the high-pressure study of paracelsian, BaAl_2_Si_2_O_8_, revealed three new phase transitions with the formation of three previously unknown high-pressure polymorphs, paracelsian-II, paracelsian-III and paracelsian-IV. The I → II phase transition is associated with the formation of pentacoordinated Al^3+^ and Si^4+^ ions, which remarkably occurs in a stepwise fashion by formation of additional Al–O and Si–O bonds, so that, technically speaking, paracelsian-II can be separated into three other polymorphs, II*a*, II*b* and II*c*, with different number of additional bonds. The II → III phase transition is reconstructive and associated with the changes of Si^4+^ and Al^3+^ coordination, which show rather complex behaviour with the general tendency towards increasing coordination numbers. Finally, the III → IV transition has a displacive character. In the course of I → II → III → IV transformation pathway, the structure becomes denser: paracelsian-II is based upon elements of cubic and hexagonal close-packing arrangements of large O^2−^ and Ba^2+^ ions, whereas, in the crystal structure of paracelsian-III and IV, this arrangement corresponds to 9-layer closest-packing with the layer sequence **ABACACBCB**.

## Supplementary information

Dataset 1

Dataset 2

## Data Availability

All data generated or analysed during this study are included in this published article (and its Supplementary Information files).
