# Supplementary material for: High pressure phase transitions of paracelsian BaAl2Si2O8
Source: Sci Rep. 2019 Sep 2;9:12652. doi: 10.1038/s41598-019-49112-1 (PMC6718520; doi:10.1038/s41598-019-49112-1)
Supplement: Supplementary file 1 — Dataset 1 [file 41598_2019_49112_MOESM1_ESM.pdf]

High pressure phase transitions of paracelsian $BaAl_2Si_2O_8$

Liudmila A. Gorelova,[†] *Anna S. Pakhomova,*[§] *Sergey V. Krivovichev,*[,†,⊦,*]

Leonid S. Dubrovinsky,[⊥] *Anatoly V. Kasatkin*[#]

Table S1. Crystallographic data and refinement parameters for paracelsian polymorphs from the experiment.

Crystal data	0.0001 GPa	0.14 GPa	3.01 GPa	6.84 GPa	10.66 GPa	14.22 GPa
	Paracelsian-I			Paracelsian-II		
Space group				$P2_1/c$		
a, Å	8.5756(1)	8.5663(2)	8.5385(2)	8.9406(3)	8.9359(2)	8.8971(3)
b, Å	9.5731(3)	9.5654(6)	9.4721(5)	9.0353(6)	8.9109(4)	8.8374(5)
c, Å	9.0681(3)	9.0579(5)	8.8015(5)	7.1777(6)	6.9954(5)	6.9042(5)
β, °	90.1696(17)	90.183(3)	90.185(3)	90.243(4)	90.041(4)	90.095(4)
Volume, Å^3	744.44(4)	742.21(7)	711.85(6)	579.81(6)	557.02(5)	542.86(5)
Z				4		
Data collection						
Wavelength, Å				0.2905		
Max. θ°	18.335	18.069	18.131	18.044	18.153	17.789
Index ranges	$-14\leq h\leq 14$	$-17\leq h\leq 16$	$-17\leq h\leq 16$	$-17\leq h\leq 18$	$-17\leq h\leq 18$	$-17\leq h\leq 18$
	$-16\leq k\leq 15$	$-15\leq k\leq 14$	$-15\leq k\leq 14$	$-15\leq k\leq 14$	$-14\leq k\leq 15$	$-14\leq k\leq 15$
	$-12\leq l\leq 15$	$-13\leq l\leq 14$	$-13\leq l\leq 14$	$-11\leq l\leq 10$	$-9\leq l\leq 10$	$-9\leq l\leq 10$
No.meas.refl.	4645	3167	3008	2449	2383	2277
No.uniq.refl.	2570	1941	1866	1483	1427	1393
No. obs.refl ($I > 2\sigma(I)$)	2426	1717	1757	1400	1304	1318
Refinement of the structure						
No.of variables	119	119	119	119	119	119
R_{int}	0.0329	0.0264	0.0249	0.0205	0.0291	0.0211
R_1, all data	0.0297	0.0360	0.0297	0.0256	0.0270	0.0228
R_1, $I > 2\sigma(I)$	0.0286	0.0296	0.0275	0.0240	0.0227	0.0213
wR_2, all data	0.0844	0.1241	0.0993	0.0648	0.0599	0.0574
wR_2, $I > 2\sigma(I)$	0.0727	0.1046	0.0825	0.0633	0.0556	0.0565
GooF	1.040	0.945	0.819	1.143	1.109	1.103

Crystal data	17.76 GPa	21.25 GPa	24.70 GPa	28.50 GPa	32.42 GPa
	Paracelsian-III			Paracelsian-IV	Paracelsian-V
Space group		$P2_1/c$		$Pna2_1$	Pn
a, Å	8.8683(3)	8.8425(4)	8.8068(4)	5.434(4)	8.742(3)
b, Å	8.7814(6)	8.7323(7)	8.6895(7)	8.743(3)	5.387(3)
c, Å	6.8155(7)	6.7210(8)	6.6556(8)	9.6849(19)	9.634(2)
β, °	89.905(5)	89.868(6)	89.845(7)	90	91.35(2)
Volume, Å^3	530.76(6)	518.96(8)	509.32(8)	460.1(4)	453.5(3)
Z			4		2
Data collection					
Wavelength, Å			0.2905		
Max. θ°	17.940	17.906	18.025	11.461	17.768
Index ranges	$-18\leq h\leq 17$	$-18\leq h\leq 17$	$-18\leq h\leq 17$	$-5\leq h\leq 5$	$-14\leq h\leq 16$
	$-15\leq k\leq 14$	$-15\leq k\leq 14$	$-15\leq k\leq 14$	$-10\leq k\leq 10$	$-7\leq k\leq 6$
	$-9\leq l\leq 10$	$-9\leq l\leq 10$	$-9\leq l\leq 10$	$-13\leq l\leq 13$	$-19\leq l\leq 19$
No.meas.refl.	2251	2133	2146	790	1747
No.uniq.refl.	1349	1278	1285	602	1743
No. obs.refl ($I > 2\sigma(I)$)	1250	1167	1185	501	1200
No.of variables	119	119	109	80	136
R_{int}	0.0236	0.0207	0.0214	0.1177	0.1389
R_1, all data	0.0323	0.0360	0.0372	0.1017	0.1117
R_1, $I > 2\sigma(I)$	0.0287	0.0296	0.0338	0.0824	0.0897
wR_2, all data	0.0916	0.0934	0.1158	0.2260	0.2347
wR_2, $I > 2\sigma(I)$	0.0865	0.0816	0.1074	0.1992	0.2116
GooF	1.094	1.158	1.068	1.087	1.009

Table S2. Bond distances and polyhedral parameters in paracelsian

Pressure	**0.0001 GPa**	**0.14 GPa**	**3.01 GPa**	**6.84 GPa**	**10.66 GPa**	**14.22 GPa**	**17.76 GPa**	**21.25 GPa**	**24.70 GPa**
Phase (Sp.gr.)	**Paracelsian-I (*P*2$_1$/*c*)**			**Paracelsian-II (*P*2$_1$/*c*)**			**Paracelsian-III (*P*2$_1$/*c*)**		
CN(Si1, Si2) / CN(Al1, Al2)	**4, 4 / 4, 4**	**4, 4 / 4, 4**	**4, 4 / 4, 4**	**4, 4 / 5, 5**	**4, 4 / 5, 5**	**4, 4 / 5, 5**	**4, 5 / 5, 5**	**4, 5 / 5, 5**	**4, 5 / 5, 5**
	SiO$_n$ polyhedra								
	SiO$_4$								
**Si1–O5**	1.615(2)	1.613(4)	1.607(3)	1.634(3)	1.633(3)	1.625(3)	1.623(4)	1.620(5)	1.619(6)
**Si1–O8**	1.615(2)	1.613(4)	1.606(3)	1.615(2)	1.625(3)	1.618(2)	1.615(4)	1.609(4)	1.604(5)
**Si1–O1**	1.627(2)	1.637(4)	1.630(3)	1.622(2)	1.616(3)	1.615(2)	1.615(4)	1.608(5)	1.605(5)
**Si1–O4**	1.632(2)	1.625(4)	1.623(3)	1.615(2)	1.610(3)	1.606(2)	1.602(4)	1.601(5)	1.597(5)
**Si1–O4**	4.199(2)	4.192(5)	3.964(4)	2.755(3)	2.628(3)	2.576(3)	2.530(4)	2.490(5)	2.449(5)
**<Si1–O>**	1.622	1.622	1.616	1.622	1.621	1.616	1.614	1.609	1.606
**Volume**	2.181	2.181	2.157	2.179	2.171	2.149	2.137	2.116	2.101
	SiO$_4$						***SiO$_5$***		
**Si2–O6**	1.604(1)	1.600(3)	1.600(3)	1.614(2)	1.612(3)	1.605(2)	1.606(4)	1.603(5)	1.599(5)
**Si2–O7**	1.622(2)	1.625(4)	1.619(3)	1.628(2)	1.629(3)	1.624(2)	1.622(4)	1.624(5)	1.620(5)
**Si2–O3**	1.637(2)	1.634(4)	1.631(3)	1.642(3)	1.645(3)	1.646(3)	1.643(4)	1.651(4)	1.653(4)
**Si2–O2**	1.640(2)	1.642(4)	1.630(3)	1.608(3)	1.603(3)	1.623(3)	1.652(4)	1.684(5)	1.706(6)
**Si2–O2**	3.972(2)	3.957(5)	3.757(5)	2.634(3)	2.474(3)	2.346(3)	2.208(4)	2.077(5)	1.993(5)
**<Si2–O>**	1.626	1.625	1.620	1.623	1.622	1.625	1.746	1.728	1.714
**Volume**	2.202	2.200	2.178	2.168	2.146	2.133	4.258	4.217	4.160
	AlO$_n$ polyhedra								
	AlO$_4$			***AlO$_5$***					
**Al1–O7**	1.731(2)	1.732(4)	1.721(3)	1.800(3)	1.807(3)	1.800(3)	1.791(4)	1.783(5)	1.778(6)
**Al1–O2**	1.733(2)	1.731(4)	1.727(3)	1.702(3)	1.703(3)	1.705(3)	1.712(4)	1.721(5)	1.725(5)
**Al1–O1**	1.743(2)	1.735(4)	1.732(3)	1.749(2)	1.750(3)	1.744(2)	1.740(4)	1.736(4)	1.731(5)
**Al1–O3**	1.753(2)	1.763(4)	1.747(3)	1.775(3)	1.780(3)	1.780(3)	1.780(4)	1.772(5)	1.769(5)
**Al1–O3**	4.224(2)	4.201(5)	3.983(4)	2.279(3)	2.108(3)	2.054(3)	2.021(4)	1.996(5)	1.971(5)
**<Al1–O>**	1.740	1.740	1.732	1.861	1.830	1.817	1.809	1.801	1.795
**Volume**	2.673	2.675	2.638	5.178	5.026	4.935	4.878	4.824	4.774
	AlO$_4$			***AlO$_5$***					
**Al2–O6**	1.718(1)	1.716(3)	1.716(43	1.745(2)	1.737(3)	1.732(2)	1.722(4)	1.715(5)	1.712(5)

**Al2–O5**	1.744(2)	1.746(4)	1.735(3)	1.778(3)	1.776(3)	1.774(2)	1.773(4)	1.766(4)	1.761(5)
**Al2–O8**	1.754(2)	1.759(4)	1.747(3)	1.834(3)	1.956(4)	1.951(3)	1.935(4)	1.910(5)	1.896(6)
**Al2–O4**	1.757(2)	1.759(5)	1.749(3)	1.738(3)	1.744(3)	1.742(3)	1.743(4)	1.742(5)	1.742(5)
**Al2–O8**	4.073(2)	4.063(5)	3.857(5)	2.410(3)	2.095(4)	2.034(3)	1.990(4)	1.955(5)	1.927(5)
**<Al2–O>**	1.743	1.744	1.737	1.901	1.862	1.847	1.832	1.818	1.807
**Volume**	2.708	2.715	2.679	5.526	5.358	5.258	5.155	5.047	4.971

				BaO_n polyhedra					
	$BaO_{7\text{-}9}$			***BaO_{11}***					
**Ba–O7**	2.735(2)	2.732(4)	2.699(2)	2.882(2)	2.850(3)	2.814(2)	2.753(4)	2.691(5)	2.654(5)
**Ba–O5**	2.759(2)	2.758(4)	2.722(2)	2.807(3)	2.687(3)	2.648(2)	2.609(4)	2.574(5)	2.550(5)
**Ba–O4**	2.783(2)	2.777(4)	2.727(3)	2.745(2)	2.722(3)	2.699(2)	2.678(3)	2.658(4)	2.639(5)
**Ba–O1**	2.805(2)	2.793(5)	2.704(4)	2.724(3)	2.680(3)	2.642(3)	2.604(5)	2.577(5)	2.545(6)
**Ba–O3**	2.826(2)	2.816(4)	2.771(2)	2.945(3)	2.920(3)	2.873(3)	2.828(4)	2.772(4)	2.738(4)
**Ba–O2**	2.833(2)	2.821(4)	2.754(3)	2.761(2)	2.763(3)	2.764(2)	2.770(3)	2.781(4)	2.779(4)
**Ba–O8**	2.861(2)	2.856(4)	2.775(3)	2.891(2)	2.935(2)	2.937(2)	2.934(4)	2.939(4)	2.930(5)
**<Ba–O>**	2.800	2.793	2.736						
**Volume**	29.030	28.780	26.544						
**Ba–O3**	3.317(2)	3.323(5)	3.317(4)						
**Ba–O4**	3.324(2)	3.333(4)	3.321(4)						
**<Ba–O>**	2.916	2.910	2.866						
**Volume**	40.959	40.777	38.319						
**Ba–O5**				2.989(2)	3.014(3)	2.992(2)	2.969(4)	2.949(4)	2.924(5)
**Ba–O1**				2.816(2)	2.757(2)	2.714(2)	2.682(3)	2.649(4)	2.626(5)
**Ba–O7**				2.984(3)	2.877(3)	2.855(2)	2.869(4)	2.882(5)	2.872(5)
**Ba–O6**				2.766(2)	2.682(3)	2.638(2)	2.608(4)	2.584(5)	2.562(5)
**<Ba–O>**				2.846	2.808	2.780	2.755	2.732	2.711
**Volume**				51.382	49.056	47.459	46.067	44.801	43.745

Pressure	**28.50 GPa**	**32.42 GPa**			
Phase (Sp.gr.)	**Paracelsian-IV ($Pna2_1$)**	**Paracelsian-V (Pn)**			
CN(Si1, Si2) / CN(Al1, Al2)	**6, 6 / 6, 4+2**	**6, 6, 6, 6 / 4+2, 4+2, 6, 6**			
SiO_n polyhedra					
	SiO_6				
**Si1–O3**	1.82(5)	**Si1–O3**	1.83(4)	**Si3–O15**	1.96(3)
**Si1–O7**	1.68(6)	**Si1–O11**	2.05(3)	**Si3–O8**	1.78(3)
**Si1–O1**	1.71(12)	**Si1–O13**	1.80(4)	**Si3–O10**	1.81(3)
**Si1–O6**	1.78(4)	**Si1–O1**	1.69(4)	**Si3–O16**	1.78(4)
**Si1–O3**	2.11(5)	**Si1–O15**	1.78(3)	**Si3–O2**	1.82(3)
**Si1–O5**	1.81(6)	**Si1–O14**	1.86(3)	**Si3–O5**	1.68(3)
**<Si1–O>**	1.82	**<Si1–O>**	1.84	**<Si3–O>**	1.81
**Volume**	7.88	**Volume**	8.02	**Volume**	7.72
	SiO_6				
**Si2–O8**	1.71(6)	**Si2–O13**	1.68(3)	**Si4–O5**	1.63(4)
**Si2–O2**	1.82(5)	**Si2–O8**	1.76(3)	**Si4–O7**	1.86(3)
**Si2–O5**	1.84(5)	**Si2–O10**	1.91(3)	**Si4–O9**	1.73(4)
**Si2–O7**	1.67(5)	**Si2–O16**	1.88(4)	**Si4–O11**	1.89(3)
**Si2–O8**	1.79(6)	**Si2–O2**	1.93(3)	**Si4–O4**	1.85(3)
**Si2–O2**	1.95(5)	**Si2–O7**	1.81(2)	**Si4–O3**	2.05(4)
**<Si2–O>**	1.80	**<Si2–O>**	1.83	**<Si4–O>**	1.83
**Volume**	7.57	**Volume**	7.91	**Volume**	8.07
AlO_n polyhedra					
	AlO_6				
**Al1–O4**	1.81(5)	**Al1–O4**	1.76(3)	**Al3–O11**	1.77(3)
**Al1–O6**	1.83(5)	**Al1–O3**	1.74(3)	**Al3–O4**	1.90(3)
**Al1–O3**	1.78(6)	**Al1–O16**	1.64(4)	**Al3–O8**	1.75(3)
**Al1–O8**	1.81(6)	**Al1–O6**	1.86(3)	**Al3–O6**	1.87(3)
**Al1–O6**	1.79(5)	**Al1–O12**	1.74(3)	**Al3–O12**	1.86(3)
**Al1–O4**	1.85(5)	**Al1–O14**	1.81(3)	**Al3–O14**	1.78(3)
**<Al1–O>**	1.81	**<Al1–O>**	1.76	**<Al3–O>**	1.82
**Volume**	7.81	**Volume**	7.17	**Volume**	7.92
	AlO_6				
**Al2–O5**	1.74(6)	**Al2–O9**	1.74(4)	**Al4–O2**	1.85(3)
**Al2–O1**	1.66(11)	**Al2–O10**	1.81(3)	**Al4–O7**	1.67(3)
**Al2–O2**	1.77(5)	**Al2–O12**	1.64(3)	**Al4–O1**	1.65(4)

**Al2–O4**	1.71(5)	**Al2–O15**	1.66(3)	**Al4–O6**	1.61(3)
**Al2–O6**	2.43(6)	**Al2–O13**	2.18(4)	**Al4–O4**	2.08(3)
**Al2–O7**	2.67(7)	**Al2–O14**	2.55(4)	**Al4–O10**	2.67(3)
**<Al2–O>**	1.72	**<Al2–O>**	1.71	**<Al4–O>**	1.69
**Volume**	2.33	**Volume**	2.11	**Volume**	2.03

BaO_n polyhedra					
				BaO_{12}	
**Ba–O6**	2.55(4)	**Ba1–O16**	3.04(4)	**Ba2–O10**	2.62(3)
**Ba–O5**	2.60(6)	**Ba1–O12**	2.70(3)	**Ba2–O9**	2.65(3)
**Ba–O1**	2.49(5)	**Ba1–O5**	2.56(3)	**Ba2–O11**	2.64(3)
**Ba–O2**	2.73(4)	**Ba1–O13**	2.91(2)	**Ba2–O2**	2.80(3)
**Ba–O3**	2.52(5)	**Ba1–O14**	2.52(3)	**Ba2–O3**	2.72(3)
**Ba–O8**	2.90(5)	**Ba1–O7**	2.47(3)	**Ba2–O1**	2.56(4)
**Ba–O1**	2.67(4)	**Ba1–O10**	3.00(3)	**Ba2–O15**	2.66(3)
**Ba–O4**	2.71(4)	**Ba1–O9**	2.90(3)	**Ba2–O13**	2.64(3)
**Ba–O7**	2.59(5)	**Ba1–O11**	2.65(3)	**Ba2–O1**	2.83(4)
**Ba–O2**	2.84(5)	**Ba1–O2**	2.72(3)	**Ba2–O4**	2.59(3)
**Ba–O3**	2.73(5)	**Ba1–O3**	2.52(3)	**Ba2–O8**	2.75(2)
**Ba–O1**	2.95(5)	**Ba1–O9**	2.50(3)	**Ba2–O6**	2.63(3)
		**Ba1–O1**	2.64(4)		
**<Ba–O>**	2.688	**<Ba–O>**	2.70	**<Ba–O>**	2.68
**Volume BaO_{12}**	45.077	**Volume BaO_{13}**	47.81	**Volume BaO_{12}**	44.94

Table S3. Bond critical points coordination and distances for Si–O and Al–O bonds at different pressures.

P, GPa	0.0001	0.14	3.01	6.84	10.66	14.22	17.76	21.25	24.70	28.50	32.42	
				Si1–O5						Si1–O5	Si1–O1	Si3–O5
x	-0.443	-0.443	-0.440	-0.419	0.419	0.418	-0.418	-0.418	-0.418	0.435	-0.184	0.323
y	0.374	0.374	0.378	0.411	0.418	0.419	0.420	0.420	0.421	-0.470	0.098	0.362
z	0.172	0.173	0.177	-0.304	-0.303	-0.302	-0.303	-0.303	-0.303	0.065	-0.010	0.053
**Si–BCP (Å)**	0.671	0.671	0.670	0.677	0.677	0.674	0.674	0.673	0.672	0.734	0.695	0.692
**O–BCP (Å)**	0.942	0.942	0.940	0.957	0.956	0.951	0.950	0.948	0.946	1.078	0.997	0.993
**ρ**	0.147	0.147	0.148	0.141	0.141	0.144	0.144	0.146	0.146	0.094	0.126	0.127
**λ**	0.891	0.892	0.908	0.812	0.817	0.848	0.857	0.871	0.882	0.385	0.616	0.637
				Si1–O8						Si1–O3	Si1–O15	Si3–O16
x	-0.461	-0.461	-0.456	-0.420	0.419	0.418	-0.418	-0.418	-0.418	0.254	-0.041	0.185
y	0.481	0.482	0.486	-0.477	-0.468	-0.467	-0.465	-0.464	-0.463	-0.346	0.180	0.253
z	0.215	0.216	0.222	-0.242	-0.237	-0.236	-0.235	-0.234	-0.233	0.065	0.070	0.001
**Si–BCP (Å)**	0.672	0.670	0.668	0.671	0.673	0.671	0.670	0.668	0.667	0.736	0.722	0.719
**O–BCP (Å)**	0.944	0.940	0.938	0.944	0.951	0.947	0.945	0.941	0.938	1.081	1.055	1.056
**ρ**	0.147	0.149	0.150	0.149	0.146	0.148	0.149	0.151	0.153	0.095	0.103	0.107
**λ**	0.882	0.904	0.921	0.878	0.839	0.869	0.883	0.907	0.930	0.360	0.429	0.433
				Si1–O1						Si1–O1	Si1–O13	Si3–O8
x	-0.363	-0.363	-0.361	-0.351	0.352	0.352	-0.352	-0.352	-0.352	0.356	-0.039	0.178
y	0.414	0.414	0.415	0.431	0.436	0.437	0.437	0.438	0.438	-0.405	-0.010	0.448
z	0.256	0.257	0.266	-0.181	-0.177	-0.175	-0.174	-0.175	-0.174	-0.010	0.072	0.005
**Si–BCP (Å)**	0.676	0.676	0.675	0.674	0.672	0.672	0.672	0.669	0.668	0.700	0.731	0.722
**O–BCP (Å)**	0.954	0.955	0.951	0.948	0.944	0.943	0.943	0.938	0.936	1.007	1.074	1.058
**ρ**	0.141	0.141	0.143	0.144	0.146	0.146	0.146	0.148	0.149	0.121	0.098	0.103
**λ**	0.829	0.825	0.848	0.857	0.881	0.888	0.890	0.920	0.934	0.581	0.377	0.437

	Si1–O4									Si1–O3	Si1–O3	Si3–O10
x	-0.482	-0.482	-0.482	-0.476	0.477	0.478	-0.479	-0.479	-0.480	0.454	-0.167	0.286
y	0.394	0.394	0.397	0.428	0.066	0.436	0.437	0.438	0.439	-0.345	-0.004	0.444
z	0.288	0.288	0.294	-0.176	0.326	-0.173	-0.172	-0.172	-0.171	0.066	0.066	0.106
**Si–BCP (Å)**	0.676	0.675	0.673	0.671	0.669	0.668	0.667	0.666	0.665	0.850	0.741	0.731
**O–BCP (Å)**	0.955	0.951	0.948	0.944	0.940	0.938	0.935	0.935	0.932	1.259	1.091	1.082
**ρ**	0.141	0.143	0.145	0.147	0.149	0.150	0.152	0.152	0.153	0.050	0.093	0.097
**λ**	0.822	0.842	0.863	0.886	0.909	0.927	0.945	0.950	0.966	0.114	0.333	0.399

	Si1–O7	Si1–O14	Si3–O2
x	0.249	-0.044	0.299
y	-0.466	0.102	0.251
z	0.064	0.137	0.105
**Si–BCP (Å)**	0.692	0.744	0.735
**O–BCP (Å)**	0.992	1.114	1.088
**ρ**	0.127	0.093	0.096
**λ**	0.648	0.314	0.364

Si1–O6	Si1–O6	Si1–O11	Si3–O15
x	0.351	-0.170	0.169
y	-0.400	0.193	0.337
z	0.135	0.066	0.066
**Si–BCP (Å)**	0.720	0.825	0.784
**O–BCP (Å)**	1.059	1.225	1.173
**ρ**	0.107	0.056	0.069
**λ**	0.445	0.145	0.218

	Si2–O6									Si2–O8	Si2–O8	Si4–O5
x	0.133	0.133	0.134	0.144	-0.144	-0.145	0.145	0.146	0.146	-0.391	0.159	-0.490
y	0.314	0.315	0.315	0.305	0.306	0.307	0.306	0.306	0.306	0.155	-0.256	-0.496

z	-0.048	-0.048	-0.040	-0.475	-0.468	-0.467	-0.465	-0.462	-0.460	0.058	0.004	0.054
**Si–BCP (Å)**	0.668	0.667	0.666	0.672	0.670	0.668	0.668	0.667	0.666	0.689	0.714	0.674
**O–BCP (Å)**	0.937	0.935	0.932	0.943	0.941	0.937	0.938	0.936	0.932	0.986	1.046	0.953
**ρ**	0.149	0.150	0.152	0.147	0.148	0.150	0.150	0.151	0.153	0.131	0.111	0.144
**λ**	0.925	0.937	0.953	0.880	0.891	0.918	0.913	0.927	0.949	0.662	0.464	0.833
				**Si2–O7**						**Si2–O8**	**Si2–O7**	**Si4–O9**
x	0.011	0.010	0.010	0.016	-0.015	-0.015	0.016	0.015	0.015	-0.501	0.301	0.498
y	0.307	0.307	0.308	0.310	0.311	0.312	0.312	0.313	0.312	0.234	-0.156	-0.397
z	-0.010	-0.010	-0.006	-0.460	-0.455	-0.454	-0.454	-0.453	-0.451	0.006	0.056	-0.020
**Si–BCP (Å)**	0.674	0.676	0.673	0.675	0.675	0.674	0.673	0.674	0.672	0.699	0.734	0.707
**O–BCP (Å)**	0.948	0.953	0.946	0.953	0.954	0.951	0.949	0.951	0.948	1.013	1.078	1.027
**ρ**	0.144	0.142	0.145	0.144	0.144	0.146	0.147	0.146	0.147	0.121	0.096	0.117
**λ**	0.858	0.834	0.871	0.825	0.821	0.840	0.850	0.841	0.862	0.578	0.378	0.503
				**Si2–O3**						**Si2–O5**	**Si2–O16**	**Si4–O4**
x	0.052	0.053	0.056	0.072	-0.074	-0.075	0.076	0.077	0.078	-0.413	0.166	-0.354
y	0.234	0.234	0.232	0.214	0.212	0.210	0.207	0.204	0.202	0.318	-0.049	-0.385
z	-0.093	-0.093	-0.088	0.470	0.476	0.477	0.479	0.481	0.483	0.059	0.001	0.129
**Si–BCP (Å)**	0.678	0.677	0.676	0.680	0.681	0.681	0.680	0.682	0.683	0.745	0.752	0.741
**O–BCP (Å)**	0.958	0.956	0.955	0.962	0.965	0.965	0.963	0.968	0.970	1.100	1.126	1.107
**ρ**	0.140	0.141	0.141	0.139	0.137	0.137	0.138	0.135	0.135	0.088	0.087	0.094
**λ**	0.796	0.807	0.819	0.778	0.770	0.771	0.783	0.762	0.759	0.339	0.269	0.336
				**Si2–O2**						**Si2–O2**	**Si2–O10**	**Si4–O7**
x	0.036	0.035	0.040	0.068	-0.069	-0.070	0.071	0.072	0.073	-0.497	0.269	-0.491
y	0.346	0.346	0.344	0.324	0.320	0.316	0.310	0.305	0.302	0.232	-0.259	-0.303
z	-0.120	-0.120	-0.120	0.410	0.414	0.413	0.413	0.415	0.417	0.106	0.105	0.057
**Si–BCP (Å)**	0.679	0.681	0.677	0.669	0.667	0.673	0.682	0.692	0.699	0.735	0.760	0.748
**O–BCP (Å)**	0.960	0.965	0.955	0.939	0.937	0.950	0.971	0.992	1.007	1.087	1.146	1.109

**ρ**	0.139	0.137	0.141	0.147	0.150	0.144	0.135	0.126	0.119	0.094	0.081	0.086
**λ**	0.788	0.765	0.822	0.926	0.949	0.869	0.765	0.666	0.609	0.380	0.272	0.321

								**Si2–O2**		**Si2–O2**	**Si2–O2**	**Si4–O11**
x								0.071	0.073	-0.299	0.281	-0.366
y								0.229	0.230	0.248	-0.052	0.499
z								-0.382	-0.380	0.105	0.105	0.060
**Si–BCP (Å)**								1.237	1.191	0.775	0.767	0.761
**O–BCP (Å)**								0.840	0.802	1.175	1.162	1.131
**ρ**								0.052	0.062	0.074	0.080	0.083
**λ**								0.143	0.200	0.235	0.238	0.259

										**Si2–O8**	**Si2–O13**	**Si4–O3**
x										0.298	0.150	-0.363
y										-0.246	-0.150	-0.297
z										0.506	0.066	0.062
**Si–BCP (Å)**										0.723	0.689	0.826
**O–BCP (Å)**										1.063	0.987	1.227
**ρ**										0.103	0.131	0.057
**λ**										0.415	0.660	0.139

	**Al1–O2**									**Al1–O6**	**Al1–O16**	**Al3–O8**
x	0.041	0.041	0.045	-0.073	0.074	0.075	0.076	-0.077	-0.078	0.014	0.001	-0.011
y	-0.493	-0.494	-0.498	-0.469	-0.462	-0.457	0.452	-0.448	-0.446	-0.192	-0.120	0.386
z	-0.210	-0.211	-0.217	-0.239	-0.233	-0.230	0.228	-0.226	-0.225	0.250	0.330	0.332
**Al–BCP (Å)**	0.991	0.989	0.987	0.732	0.732	0.732	0.734	0.736	0.737	0.770	0.713	0.749
**O–BCP (Å)**	0.742	0.741	0.740	0.969	0.971	0.973	0.978	0.985	0.988	1.060	0.926	1.002
**ρ**	0.099	0.100	0.100	0.106	0.106	0.106	0.105	0.103	0.102	0.078	0.121	0.091
**λ**	0.662	0.670	0.680	0.751	0.750	0.748	0.730	0.710	0.700	0.499	0.969	0.638

				Al1–O7						Al1–O4	Al1–O3	Al3–O11
x	0.064	0.064	0.067	-0.077	0.079	0.079	-0.080	-0.080	-0.080	0.018	-0.150	-0.158
y	-0.376	-0.377	-0.379	0.409	0.415	0.418	0.422	0.425	0.427	-0.309	-0.129	0.366
z	-0.158	-0.158	-0.163	-0.279	-0.273	-0.273	-0.275	-0.278	-0.279	0.253	0.179	0.178
**Al–BCP (Å)**	0.742	0.742	0.740	0.764	0.766	0.764	0.761	0.758	0.756	0.767	0.741	0.751
**O–BCP (Å)**	0.989	0.990	0.984	1.037	1.041	1.037	1.030	1.025	1.022	1.045	0.995	1.021
**ρ**	0.099	0.098	0.100	0.083	0.081	0.083	0.085	0.087	0.088	0.079	0.098	0.092
**λ**	0.668	0.661	0.686	0.521	0.507	0.521	0.539	0.555	0.565	0.515	0.692	0.599

				Al1–O1						Al1–O8	Al1–O12	Al3–O12
x	0.151	0.151	0.152	-0.161	0.162	0.162	-0.162	-0.162	-0.162	0.131	-0.014	-0.019
y	-0.417	-0.418	-0.418	0.436	0.441	0.442	0.444	0.445	0.446	-0.256	-0.229	0.481
z	-0.252	-0.253	-0.262	-0.163	-0.156	-0.154	-0.154	-0.155	-0.155	0.333	0.258	0.258
**Al–BCP (Å)**	0.746	0.747	0.743	0.747	0.747	0.746	0.745	0.743	0.741	0.767	0.743	0.779
**O–BCP (Å)**	0.996	0.998	0.992	1.002	1.002	0.999	0.996	0.993	0.990	1.040	0.994	1.076
**ρ**	0.095	0.095	0.097	0.095	0.094	0.096	0.096	0.097	0.098	0.079	0.096	0.073
**λ**	0.640	0.634	0.658	0.629	0.630	0.645	0.656	0.668	0.682	0.518	0.685	0.444

				Al1–O3						Al1–O3	Al1–O4	Al3–O4
x	0.022	0.021	0.020	-0.021	0.020	0.020	-0.019	-0.018	-0.018	0.124	-0.141	-0.144
y	-0.394	-0.394	-0.397	0.428	0.434	0.436	0.439	0.442	0.443	-0.250	-0.224	0.486
z	-0.287	-0.288	-0.294	-0.137	-0.130	-0.130	-0.132	-0.133	-0.134	0.177	0.250	0.250
**Al–BCP (Å)**	0.749	0.752	0.745	0.755	0.756	0.756	0.756	0.754	0.752	0.754	0.749	0.796
**O–BCP (Å)**	1.006	1.012	0.997	1.021	1.024	1.024	1.024	1.019	1.017	1.024	1.012	1.108
**ρ**	0.093	0.091	0.097	0.090	0.088	0.088	0.088	0.090	0.090	0.900	0.091	0.063
**λ**	0.614	0.592	0.646	0.573	0.568	0.572	0.573	0.594	0.602	0.592	0.650	0.389

						Al1–O3				Al1–O6		
x				-0.071	0.077	0.079	-0.080	-0.081	-0.082	0.225		
y				-0.479	-0.476	-0.475	-0.474	-0.474	-0.474	-0.307		

z				-0.091	-0.087	-0.085	-0.084	-0.083	-0.082	0.249		
**Al–BCP (Å)**				0.931	0.863	0.845	0.833	0.823	0.815	0.758		
**O–BCP (Å)**				1.352	1.245	1.210	1.189	1.172	1.156	1.030		
**ρ**				0.026	0.038	0.044	0.048	0.051	0.055	0.085		
**λ**				0.113	0.198	0.237	0.266	0.291	0.316	0.581		

										**Al1–O4**		
x										0.226		
y										-0.190		
z										0.254		
**Al–BCP (Å)**										0.775		
**O–BCP (Å)**										1.071		
**ρ**										0.076		
**λ**										0.460		

	**Al2–O6**									**Al2–O5**	**Al2–O12**	**Al4–O1**
x	0.345	0.344	0.342	0.337	-0.337	-0.337	0.338	0.337	0.337	-0.345	0.133	-0.352
y	0.317	0.318	0.318	0.299	0.295	0.296	0.298	0.299	0.301	0.443	-0.432	-0.086
z	-0.046	-0.045	-0.038	-0.476	-0.462	-0.460	-0.460	-0.460	-0.461	0.162	0.237	0.298
**Al–BCP (Å)**	0.738	0.737	0.738	0.746	0.743	0.742	0.739	0.736	0.735	0.741	0.709	0.714
**O–BCP (Å)**	0.979	0.977	0.979	0.999	0.993	0.991	0.983	0.978	0.977	0.999	0.928	0.929
**ρ**	0.101	0.102	0.102	0.096	0.098	0.099	0.101	0.103	0.104	0.100	0.128	0.121
**λ**	0.701	0.711	0.705	0.629	0.650	0.663	0.692	0.713	0.723	0.676	0.981	0.950

	**Al2–O5**									**Al2–O1**	**Al2–O15**	**Al4–O7**
x	0.479	0.479	0.479	0.481	-0.482	-0.482	0.483	0.482	0.483	-0.324	0.130	-0.471
y	0.189	0.189	0.188	0.310	0.308	0.308	0.308	0.308	0.309	0.423	0.431	-0.100
z	-0.497	-0.497	-0.494	-0.469	-0.458	-0.457	-0.457	-0.458	-0.457	0.298	0.161	0.158
**Al–BCP (Å)**	0.747	0.748	0.743	0.756	0.755	0.755	0.745	0.752	0.750	0.719	0.715	0.719
**O–BCP (Å)**	0.999	1.001	0.991	1.022	1.021	1.020	0.998	1.015	1.012	0.940	0.942	0.950

**ρ**	0.094	0.094	0.098	0.089	0.090	0.090	0.090	0.091	0.092	0.117	0.121	0.117
**λ**	0.628	0.623	0.662	0.560	0.566	0.571	0.576	0.591	0.603	0.890	0.913	0.887
				**Al2–O8**						**Al2–O4**	**Al2–O9**	**Al4–O2**
x	0.456	0.455	0.451	0.418	-0.417	-0.416	0.416	0.415	0.415	-0.214	0.280	-0.491
y	0.141	0.141	0.144	0.319	0.308	0.307	0.306	0.306	0.305	0.514	0.463	0.011
z	0.381	0.381	0.381	0.396	0.407	0.408	0.408	0.408	0.407	0.239	0.286	0.210
**Al–BCP (Å)**	0.749	0.751	0.747	0.774	0.814	0.813	0.808	0.800	0.795	0.733	0.744	0.776
**O–BCP (Å)**	1.005	1.008	1.000	1.060	1.142	1.139	1.128	1.111	1.101	0.977	0.992	1.073
**ρ**	0.093	0.092	0.094	0.076	0.055	0.056	0.058	0.061	0.064	0.107	0.096	0.076
**λ**	0.610	0.600	0.628	0.462	0.303	0.309	0.329	0.360	0.379	0.740	0.664	0.466
				**Al2–O4**						**Al2–O2**	**Al2–O10**	**Al4–O4**
x	0.440	0.439	0.437	0.424	-0.423	-0.423	0.421	0.421	0.420	-0.220	0.255	-0.329
y	0.266	0.266	0.268	0.202	0.194	0.193	0.192	0.192	0.192	0.370	-0.453	-0.181
z	0.406	0.406	0.411	0.447	0.459	0.460	0.461	0.461	0.462	0.211	0.204	0.233
**Al–BCP (Å)**	0.750	0.751	0.748	0.743	0.746	0.745	0.745	0.745	0.745	0.750	0.763	0.852
**O–BCP (Å)**	1.006	1.009	1.003	0.995	0.999	0.998	0.998	0.998	0.998	1.017	1.044	1.220
**ρ**	0.092	0.092	0.094	0.098	0.096	0.096	0.096	0.096	0.096	0.093	0.083	0.042
**λ**	0.606	0.599	0.619	0.650	0.639	0.646	0.647	0.651	0.653	0.615	0.540	0.231
							**Al2–O8**				**Al2–O13**	
x					-0.417	-0.416	0.416	0.415	0.415		0.139	
y					0.212	0.212	0.213	0.214	0.214		-0.346	
z					-0.388	-0.386	-0.386	-0.385	-0.385		0.168	
**Al–BCP (Å)**					0.859	0.839	0.824	0.813	0.804		0.893	
**O–BCP (Å)**					1.236	1.195	1.165	1.142	1.122		1.288	
**ρ**					0.040	0.046	0.051	0.056	0.059		0.033	
**λ**					0.191	0.235	0.273	0.308	0.340		0.158	
